# Prognostic consistency of oncotype DX® molecular assay across racial groups in early-stage hormone receptor-positive breast cancer

**DOI:** 10.1038/s41523-026-00996-z

**Published:** 2026-06-25

**Authors:** Reine Abou Zeidane, Benjamin Hauk, Samuel Lichtman-Mikol, Courtney Pisano, Yilun Sun, Priyanka Shailendra Rana, Citlally Lopez-Flores, Breanna N. McBean, Kassidy M. Jungles, Trisha Lal, Nihit Mehta, Philip Bomeisl, Amanda L. Amin, Alberto J. Montero, Janice Lyons, Corey W. Speers

**Affiliations:** 1 Case Western Reserve University School of Medicine, Cleveland, OH USA; 2 Department of Radiation Oncology, University Hospitals Seidman Cancer Center, Cleveland, OH USA; 3 University of Nevada School of Medicine, Las Vegas, NV USA; 4 University of Michigan School of Medicine, Ann Arbor, MI USA; 5 Department of Surgery, Division of Surgical Oncology, University Hospitals Cleveland Medical Center, Cleveland, OH USA; 6 Department of Family Medicine, University Hospitals Cleveland Medical Center, Cleveland, OH USA; 7 Department of Pathology, University Hospitals Cleveland Medical Center, Cleveland, OH USA; 8 Department of Hematology and Oncology, University Hospitals Cleveland Medical Center, Cleveland, OH USA; 9 Department of Radiation Oncology, University of Alabama-Birmingham, O’Neal Cancer Center, Birmingham, AL USA

**Keywords:** Cancer, Oncology, Risk factors

## Abstract

In this retrospective cohort study, we evaluated the consistency of prognostic outcomes provided by the Oncotype DX® assay across different racial groups among women with early-stage hormone receptor-positive (HR + ) breast cancer. Data was abstracted from the electronic medical record of 1122 patients with non-metastatic HR+ breast cancer who underwent Oncotype DX testing at University Hospitals Seidman Cancer Center between 2013 and 2023. Recurrence Score (RS) categories were defined using historical cutpoints: low(0–18), intermediate(19–30), and high(31–100). Two contemporary-cutpoint sensitivity analyses based on TAILORx and RxPONDER were also performed: binary scheme (0–25;26–100) and three-level scheme (RS 0–15;16–25;26–100). Recurrence-free survival (RFS) and overall survival (OS) were analyzed using Kaplan-Meier curves and adjusted Cox models. The cohort included 142 African American (AA) and 967 White women. Under the historical cutpoints, AA women had a higher prevalence of intermediate and high-risk RS. Despite these differences, 5-year RFS and OS were comparable. In multivariable analyses, AA race was not independently associated with RFS or OS. Sensitivity analyses using contemporary cutpoints yielded concordant findings. These findings support consistent prognostic value of Oncotype DX assay across racial groups, although further investigation into socioeconomic and clinical contributors to disparities is warranted.

## Introduction

Breast cancer, the most common non-skin cancer among women worldwide, predominantly presents as hormone receptor-positive (HR + ) and human epidermal growth factor receptor 2-negative (HER2-negative)^1^. This subtype accounts for 70% of all breast cancer cases diagnosed in the United States and is traditionally managed with surgery followed by adjuvant systemic therapy including endocrine treatment and chemotherapy^2–5^. While effective, these treatments come with a range of potential side effects and can significantly impact the patient’s quality of life^6,7^. Precision medicine has advanced through the development of genomic assays including the Oncotype DX Breast Recurrence® assay. These tests have become pivotal in customizing treatment approaches, allowing for more personalized care by predicting the likelihood of cancer recurrence and the potential benefit of chemotherapy^8^.

The Oncotype DX assay is designed to guide treatment decisions in patients with early-stage HR + , HER2- breast cancer^9^. It offers prognostic insights estimating the likelihood of distant recurrence over 10 years in the natural course of disease^10^. It also provides predictive insights into identifying patients most likely to benefit from adjuvant chemotherapy, helping to avoid unnecessary treatments and their associated side effects for those with a low likelihood of benefit^11,12^. A higher score indicates a higher likelihood of cancer recurrence and more benefit from the addition of chemotherapy^13^. By providing a Recurrence Score® result between 0 and 100, this assay has significantly shaped the personalized management of breast cancer worldwide, helping clinicians tailor treatment decisions based on a tumor molecular profile^14^.

Despite advances in early detection and treatment, breast cancer outcomes continue to vary significantly, with considerable treatment and prognosis heterogeneity driven by a complex interplay of biological, clinical, and socio-environmental factors among various populations^15^. Disparities in breast cancer have long been documented^16–18^, particularly among African American (AA) patients who experience higher mortality rates, more aggressive tumor subtypes, limited access to advanced treatment modalities, and lower health-related quality of life compared to White populations^19,20^. This heterogeneity raises important questions about the equitable application of prognostic tools like the Oncotype DX assay, which has been widely adopted to guide adjuvant treatment decisions in early-stage HR + , HER2- breast cancer. Therefore, the question of how genomic tests, like the Oncotype DX assay, perform across diverse racial groups or whether specific variation might influence its predictive accuracy and clinical utility remains incompletely answered.

The current study explores the performance of the Oncotype DX assay across different racial groups to assess whether the test offers consistent prognostic value. It emphasizes the influence of tumor biology and clinical factors on shaping the prognostic significance. Understanding these dynamics is essential for advancing equitable, evidence-based care for all patients with breast cancer and ensuring that the Oncotype DX assay fulfills its promise of personalized medicine for all patients regardless of race. Addressing these disparities will not only enhance the overall effectiveness and inclusivity of breast cancer care strategies worldwide, but also help inform targeted real-world, community-based interventions to improve breast cancer outcomes for underserved populations.

## Results

### Baseline patient characteristics and Oncotype DX risk distribution

**Fig. 1:**
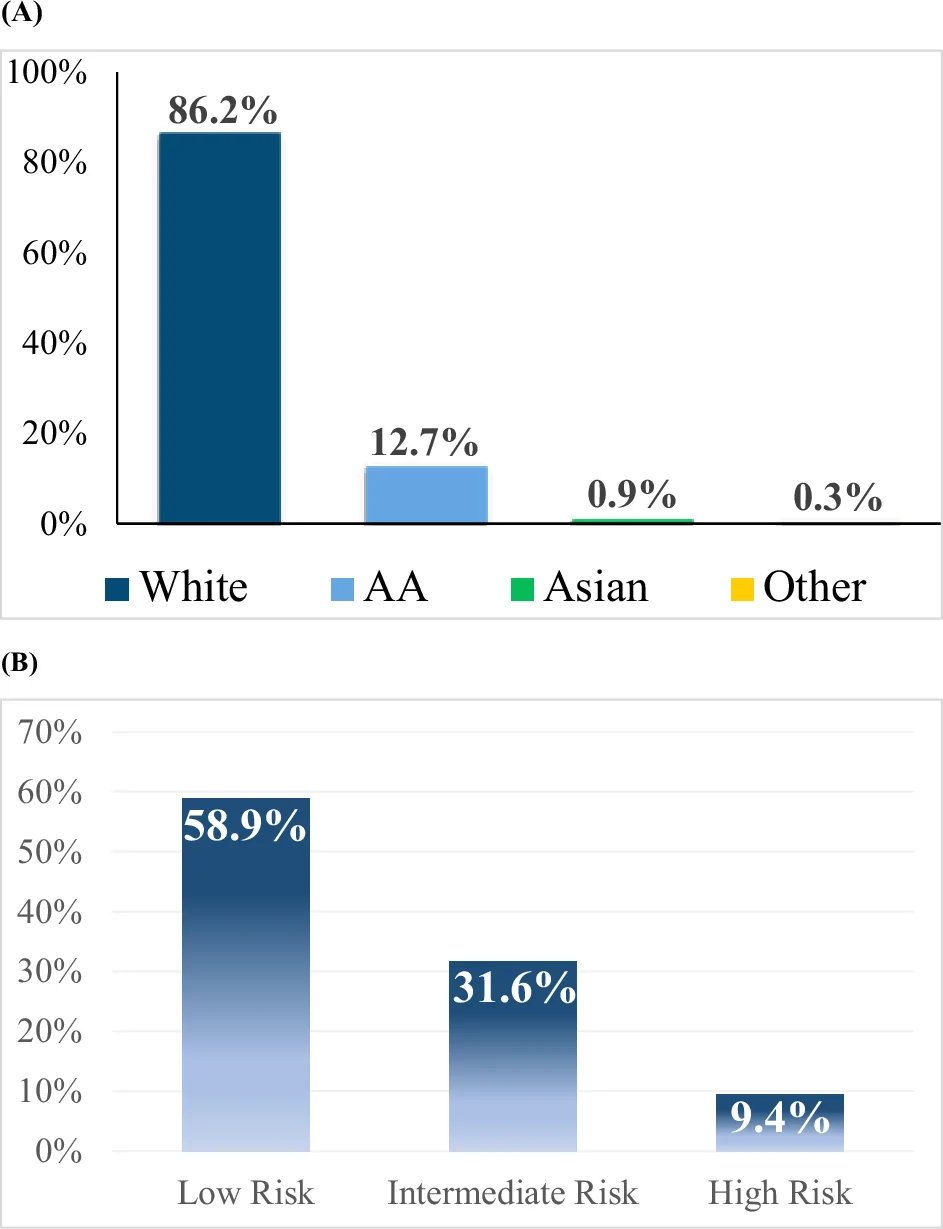
Racial distribution of Oncotype DX testing and historical Recurrence Score risk categories. **A** Distribution of patients who underwent Oncotype DX testing across racial groups. **B** Distribution of Oncotype DX Recurrence Score risk categories across racial groups using historical cutpoints: low risk, RS 0-18; intermediate risk, RS 19-30; and high risk, RS 31-100. Contemporary-cutpoint distributions are presented in Tables 5 and  7.

A total of 1122 women with non-metastatic breast cancer and Oncotype DX results were included in this institutional study with a demographic breakdown of the entire cohort depicted in Table S1. The mean age at diagnosis was 60.8 ± 10.5 with a median of 61.7 years (IQR: 32–96). Among these patients, 142 (12.7%) identified as African American (AA), 967 (86.2%) as White, 10 (0.9%) as Asian, and 3 (0.3%) as belonging to other racial groups, as shown in Fig. 1A. Using the historical Recurrence Score categories, 661 (58.9%) patients had low-risk scores, 355 (31.6%) had intermediate-risk scores, and 106 (9.4%) had high-risk scores as illustrated in Fig. 1B. For the contemporary-cutpoint sensitivity analyses, numeric RS values were available for 1111 patients. With RS 0-25 defined as low risk and RS 26-100 defined as high risk, 920 (82.8%) patients were low risk and 191 (17.2%) were high risk. AA women had a numerically higher frequency of high-risk RS 26–100 than White women (20.1% vs. 16.8%). Using the contemporary three-level schema, 512 (46.1%) patients had RS 0–15, 408 (36.7%) had RS 16–25, and 191 (17.2%) had RS 26–100.

The mean Oncotype DX score was 18.6 for AA women and 17.9 for White women (*P* = 0.4), indicating no statistically significant difference. AA women had a higher proportion of intermediate-risk scores (34.5%) compared to White women (31.3%) and a greater proportion of high-risk Oncotype DX results (12.7%) compared to White women (9.0%). A detailed breakdown of Recurrence Score results stratified by racial groups is presented in Table 1, providing further insights into the distribution of genomic risk among the study population.

**Table 1:** Recurrence Score results stratified by racial group

Oncotype DX	White	AA	Asian	Other	Overall
	*N* = 967	*N* = 142	*N* = 10	*N* = 3	*N* = 1122
Low Risk	577 (59.7%)	75 (52.8%)	7 (70.0%)	2 (66.7%)	661 (58.9%)
Intermediate Risk	303 (31.3%)	49 (34.5%)	2 (20.0%)	1 (33.3%)	355 (31.6%)
High Risk	87 (9.0%)	18 (12.7%)	1 (10.0%)	0 (0%)	106 (9.4%)

(historical cutpoints: low RS 0–18, intermediate RS 19–30, high RS 31–100).

### Survival analysis

The median follow-up time was 5.5 years (95% CI: 5.28–5.78). As outlined in Table S2, the 5-year OS rates were similar between AA (94.0%;[89.3–98.8]) and White women (93.2%; [91.4–95.1%]). The 10-year OS rates showed a slight advantage for AA women (78.2% [65.7–93.1]) as compared to White women (74.8% [68.4–81.8]). Similarly, the 5-year RFS rates were nearly identical, at 90.7% [85.2–96.5] for AA women and 90.4% [88.3–92.6] for White women. The 10-year RFS rates showed a modest difference, with AA women having a rate of 76.8% [65.9–89.5] compared to 74.6% [68.5–81.2] for White women.

Multivariable Cox proportional hazards modeling showed no significant differences in RFS between AA and White women (HR = 1.25, 95% CI: 0.72–2.19; *P* = 0.43) after adjusting for Oncotype DX risk groups and clinical factors. In addition, there were no significant differences in OS (HR = 1.12, 95% CI: 0.57–2.21; *P* = 0.74) after adjusting for Oncotype DX risk groups and other clinical characteristics.

Several factors were associated with worse RFS and OS and these include higher risk levels (defined as intermediate and high Recurrence Score results), larger tumor size (T stage), nodal involvement (N0 vs. N1), and older age (Table 2). Additionally, the presence of diabetes was associated with worse OS (*P* = 0.03) but not with worse RFS (*P* = 0.16). Importantly, race was not independently associated with differences in RFS or OS at any time point in any of the analyses.

**Table 2:** Multivariable Cox proportional hazards model analysis for RFS and OS using historical cutpoints: low risk RS 0–18, intermediate risk RS 19–30, and high risk RS 31–100

	RFS			OS		
*Predictors*	*HR*	*95% CI*	*P value*	*HR*	*95% CI*	*P value*
White	*Reference*			*Reference*		
AA	1.25	0.72–2.19	0.426	1.12	0.57–2.21	0.735
Low Risk	*Reference*			*Reference*		
Intermediate Risk	1.72	1.16–2.55	**0.007**	2.08	1.34–3.25	**0.001**
High Risk	1.42	0.73–2.73	0.298	1.97	0.97–3.99	0.061
T1-	*Reference*			*Reference*		
T2+	1.93	1.32–2.81	**0.001**	1.46	0.95–2.25	0.086
N0	*Reference*			*Reference*		
N1+	1.99	1.21–3.27	**0.006**	1.88	1.08–3.30	**0.027**
Age	1.06	1.03–1.08	**<0.001**	1.08	1.06–1.11	**<0.001**
Cardiac disease	1.28	0.80–2.06	0.305	1.35	0.79–2.30	0.266
Diabetes	1.60	0.83–3.07	0.162	2.07	1.07–4.04	**0.032**
Obesity	1.04	0.33–3.30	0.951	0.99	0.24–4.07	0.984
Observations	1063			1063		

Bold indicates *p* values ≤ 0.07.

Subgroup analysis by Recurrence Score results further revealed that the 5-year RFS rates were similar for low-risk patients, with AA women showing a rate of 97.4% compared to 92.1% for White women. Among high-risk patients, the 5-year RFS rates were 92.9% for AA women and 86.9% for White women. However, a notable trend emerged in the intermediate-risk group, where AA women exhibited worse RFS rates. Specifically, the 5-year RFS rates for intermediate-risk AA women were 80.5% compared to 88.1% for White women as depicted in Table 3. An interaction model demonstrated a trend toward worse RFS for intermediate-risk AA women (HR = 1.99, 95% CI: 0.95-4.18; *P* = 0.07). However, the wide confidence interval suggests limited precision (Table 4).

**Table 3:** RFS and OS stratified by Recurrence Score results

Oncotype DX		Race	Low Risk	Intermediate Risk	High Risk
RFS	3-year	AA	100 (100–100)	86.7 (77.3–97.2)	92.9 (80.3–100)
		White	95.7 (94.0–97.5)	94.1 (91.3–97.1)	93.4 (88.0–99.2)
	5-year	AA	97.4 (92.6–100)	80.5 (69.0–94.0)	92.9 (80.3–100)
		White	92.1 (89.6–94.7)	88.1 (83.9–92.5)	86.9 (79.1–95.4)
	10-year	AA	83.4 (68.7–100)	67.8 (50.3–91.3)	77.4 (52.6–100)
		White	79.1 (70.4–88.9)	72.3 (64.4–81.1)	54.3 (34.0–86.7)
OS	3-year	AA	100 (100–100)	95.5 (89.5–100)	100 (100–100)
		White	98.3 (97.2–99.5)	97.1 (95.0–99.2)	94.6 (89.6–99.9)
	5-year	AA	97.4 (92.6–100)	86.8 (76.6–98.5)	100 (100–100)
		White	95.5 (93.5–97.5)	91.1 (87.3–95.0)	86.1 (77.9–95.1)
	10-year	AA	78.5 (60.8–100)	74.4 (53.7–100)	83.3 (58.3–100)
		White	79.2 (69.7–89.9)	72.5 (64.5–81.6)	53.8 (33.7–86.0)

(historical cutpoints: low RS 0–18, intermediate RS 19–30, high RS 31–100).

**Table 4:** Multivariable Cox proportional hazards model analysis with interaction with race (historical cutpoints: low RS 0–18, intermediate RS 19–30, high RS 31–100)

Endpoint	Oncotype DX	HR (95% CI), AA vs. White	*P* value
RFS	Low Risk	0.65 (0.23–1.82)	0.42
	Intermediate Risk	1.99 (0.95–4.18)	**0.07**
	High Risk	1.56 (0.33–7.40)	0.57
OS	Low Risk	0.98 (0.34–2.78)	0.97
	Intermediate Risk	1.39 (0.53–3.64)	0.50
	High Risk	0.82 (0.10–6.66)	0.85

Bold indicates *p* values ≤ 0.07.

Sensitivity analyses using contemporary Recurrence Score cutpoints produced similar conclusions (Tables 5–10). In the binary RS 0-25 versus RS 26–100 model, AA race was not associated with RFS (HR = 1.33; 95% CI: 0.77-2.28; *P* = 0.302) or OS (HR = 1.24; 95% CI: 0.65–2.36; *P* = 0.519) (Table 9). High-risk RS 26-100 was associated with the expected adverse direction for RFS (HR = 1.42; 95% CI: 0.92–2.19; *P* = 0.113) and OS (HR = 1.59; 95% CI: 0.98–2.56; *P* = 0.058). The race-by-high-risk RS interaction was not significant for RFS (interaction HR = 1.69; 95% CI: 0.52–5.45; *P* = 0.382) or OS (interaction HR = 0.60; 95% CI: 0.12–3.02; *P* = 0.532), supporting the conclusion that the prognostic performance of the assay was broadly consistent across AA and White women in this cohort. A three-level contemporary sensitivity analysis using RS 0–15, 16–25, and 26–100 also demonstrated no independent association between AA race and RFS or OS (Table 10).

**Table 5:** Contemporary binary Recurrence Score cutpoint distribution (RS 0–25 vs RS 26–100) by racial group (sensitivity analysis)

RS category	White, *N* = 954	AA, *N* = 139	Overall, *N* = 1093
Low Risk (RS 0–25)	793 (83.1%)	111 (79.9%)	904 (82.7%)
High Risk (RS 26–100)	161 (16.9%)	28 (20.1%)	189 (17.3%)

**Table 6:** RFS and OS estimates stratified by contemporary binary Recurrence Score cutpoint (RS 0–25 vs RS 26–100) and race

Endpoint	Time	Race	Low (RS 0–25)	High (RS 26–100)
RFS	3-yr	AA	95.6 (88.7–98.3)	88.2 (67.6–96.1)
		White	95.2 (93.3–96.6)	94.5 (89.3–97.2)
	5-yr	AA	92.7 (84.3–96.7)	81.4 (56.2–92.9)
		White	90.9 (88.2–92.9)	90.0 (83.3–94.1)
	10-yr	AA	78.0 (60.8–88.3)	71.2 (40.0–88.2)
		White	77.7 (69.9–83.7)	65.5 (46.7–79.1)
OS	3-yr	AA	97.8 (91.6–99.5)	100 (100–100)
		White	97.8 (96.4–98.7)	97.2 (92.6–98.9)
	5-yr	AA	93.5 (85.0–97.3)	95.7 (72.9–99.4)
		White	94.0 (91.7–95.7)	91.6 (84.8–95.4)
	10-yr	AA	76.7 (56.0–88.5)	85.0 (47.6–96.5)
		White	78.5 (70.0–84.9)	64.5 (45.9–78.2)

Values are % (95% CI).

**Table 7:** Contemporary three-level Recurrence Score cutpoint distribution (RS 0–15 / 16–25 / 26–100) by racial group (sensitivity analysis)

RS category	White, *N* = 954	AA, *N* = 139	Overall, *N* = 1093
Low Risk (RS 0–15)	445 (46.6%)	57 (41.0%)	502 (45.9%)
Intermediate Risk (RS 16–25)	348 (36.5%)	54 (38.8%)	402 (36.8%)
High Risk (RS 26–100)	161 (16.9%)	28 (20.1%)	189 (17.3%)

**Table 8:** RFS and OS estimates stratified by contemporary three-level Recurrence Score cutpoint (RS 0–15 / 16–25 / 26–100) and race

Endpoint	Time	Race	Low (RS 0–15)	Intermediate (RS 16–25)	High (RS 26–100)
RFS	3-yr	AA	100 (100–100)	91.8 (79.6–96.9)	88.2 (67.6–96.1)
		White	95.8 (93.2–97.4)	94.5 (91.2–96.6)	94.5 (89.3–97.2)
	5-yr	AA	96.0 (74.8–99.4)	89.4 (76.3–95.5)	81.4 (56.2–92.9)
		White	92.1 (88.6–94.5)	89.3 (84.8–92.5)	90.0 (83.3–94.1)
	10-yr	AA	92.0 (71.6–97.9)	68.3 (44.1–83.7)	71.2 (40.0–88.2)
		White	75.4 (60.8–85.2)	79.5 (72.2–85.1)	65.5 (46.7–79.1)
OS	3-yr	AA	100 (100–100)	95.9 (84.7–99.0)	100 (100–100)
		White	98.1 (96.1–99.1)	97.5 (94.8–98.8)	97.2 (92.6–98.9)
	5-yr	AA	96.0 (74.8–99.4)	90.8 (77.1–96.5)	95.7 (72.9–99.4)
		White	94.7 (91.5–96.7)	93.2 (89.2–95.7)	91.6 (84.8–95.4)
	10-yr	AA	82.3 (37.9–96.2)	71.4 (45.0–86.8)	85.0 (47.6–96.5)
		White	77.6 (62.3–87.3)	79.1 (69.4–86.0)	64.5 (45.9–78.2)

Values are % (95% CI).

**Table 9:** Multivariable Cox proportional hazards model sensitivity analysis using contemporary binary Recurrence Score cutpoints

	RFS			OS		
*Predictors*	*HR*	*95% CI*	*P value*	*HR*	*95% CI*	*P value*
White	*Reference*			*Reference*		
AA	1.33	0.77–2.28	0.302	1.24	0.65–2.36	0.519
Low Risk (0–25)	*Reference*			*Reference*		
High Risk (26–100)	1.42	0.92–2.19	0.113	1.59	0.98–2.56	0.058
T1-	*Reference*			*Reference*		
T2+	1.62	1.12–2.34	**0.011**	1.20	0.78–1.84	0.411
N0	*Reference*			*Reference*		
N1+	1.55	1.00–2.40	0.051	1.53	0.94–2.48	0.087
Age	1.05	1.03–1.07	**<0.001**	1.07	1.05–1.10	**<0.001**
Cardiac disease	1.25	0.79–1.98	0.350	1.29	0.77–2.15	0.335
Diabetes	1.77	0.94–3.31	0.076	2.35	1.24–4.45	**0.009**
Obesity	1.05	0.33–3.33	0.934	1.04	0.25–4.25	0.961

Bold indicates *p* values ≤ 0.07.

**Table 10:** Multivariable Cox proportional hazards model sensitivity analysis using contemporary three-level Recurrence Score categories

	RFS			OS		
*Predictors*	*HR*	*95% CI*	*P value*	*HR*	*95% CI*	*P value*
White	*Reference*			*Reference*		
AA	1.31	0.76–2.26	0.324	1.22	0.64–2.32	0.556
Low Risk (0–15)	*Reference*			*Reference*		
Intermediate Risk (16–25)	1.30	0.86–1.97	0.209	1.44	0.91–2.29	0.123
High Risk (26–100)	1.62	1.00–2.64	0.052	1.91	1.11–3.28	**0.019**
T1-	*Reference*			*Reference*		
T2+	1.58	1.09–2.30	**0.015**	1.17	0.76–1.80	0.482
N0	*Reference*			*Reference*		
N1+	1.55	1.00–2.41	0.050	1.55	0.95–2.51	0.079
Age	1.05	1.03–1.07	**<0.001**	1.08	1.05–1.10	**<0.001**
Cardiac disease	1.24	0.78–1.97	0.365	1.29	0.77–2.15	0.335
Diabetes	1.76	0.94–3.30	0.077	2.33	1.23–4.40	**0.009**
Obesity	1.05	0.33–3.33	0.936	1.03	0.25–4.25	0.963

Bolding indicates values with *p* value ≤ 0.07.

## Discussion

This study underscores the robust prognostic value of the Oncotype DX assay across different racial groups, specifically focusing on African American (AA) and White women. While AA women frequently exhibit higher Recurrence Score results, suggesting a predisposition to more aggressive disease, the overall long-term outcomes, including recurrence-free survival (RFS) and overall survival (OS), are largely similar between these racial groups. This highlights the complexity of cancer prognosis where genomic data alone cannot fully account for survival disparities. Our findings are consistent with studies indicating that AA women often present with higher Recurrence Score results^21–24^, potentially reflecting a greater underlying biological propensity for more aggressive disease. Notably, within the intermediate-risk category, there appears to be a trend toward poorer RFS among AA women (HR = 1.99 *P* = 0.07), though not reaching statistical significance. This finding suggests the potential influences from non-genomic factors that may include treatment response or social determinants of health, including healthcare access. Overall, however, the consistency of the Oncotype DX assay across racial boundaries, as observed in this study, supports the universal applicability of the Oncotype DX assay in breast cancer management. Further, this study supports the hypothesis that while the Oncotype DX assay provides valuable prognostic information, its interpretation and subsequent clinical decision-making must consider broader societal and clinical contexts to ensure equitable care.

Variability in findings is observed not only across institutions and racial groups but also within clinical trials. The post hoc analysis of the TAILORx trial showed that among patients with recurrence scores of 11 to 25, AA women had the highest 8-year locoregional recurrence probability compared with White patients (5.1% vs 1.8%; *P* < 0.001); however, in the subgroup with recurrence scores greater than 25, Asian patients had the highest 8-year LRR probability compared with White patients (10.6% vs 3.0%; *P* = 0.026)^25^. Furthermore, some studies have suggested that the predictive accuracy of the Recurrence Score may vary across racial and ethnic groups, with stronger prognostic performance observed in non-Hispanic White women compared to patients of African ancestry and Hispanic patients^26^, though there are important limitations that constrain its applicability. Specifically, the most frequently cited registry-based work in this area relied on the National Cancer Data Base (NCDB), which (a) captures only breast cancer–specific mortality endpoints and lacks structured recurrence data, making it difficult to validate the Recurrence Score as a true prognostic tool for recurrence; (b) provides only coarse, registry-derived treatment variables and does not capture true treatment compliance, dose intensity, or completion of endocrine therapy; and (c) provides only limited adjustment for measured confounders, with substantial residual confounding from unmeasured social determinants of health. Our study addresses these limitations by providing recurrence-based RFS endpoints in a single-institution EMR-based cohort with longer and more uniform follow-up, and by evaluating prognostic consistency using both historical and contemporary RS cutpoints. We note, however, that our dataset has its own limitations, including the absence of structured chemotherapy, radiation, endocrine therapy, and menopausal status variables, which precludes us from fully assessing how treatment differences may contribute to outcome differences. In this study there appears to be a trend toward poorer RFS among AA women within the intermediate-risk category (HR = 1.99 *P* = 0.07). In this study, the originally defined intermediate-risk category corresponded to RS 19-30, whereas the contemporary intermediate range is more commonly considered RS 16–25 for premenopausal patients and RS 0–25 as a group in postmenopausal patients when considering chemotherapy benefit. Thus, the observed trend toward poorer RFS among AA women in the original intermediate-risk category should be interpreted cautiously because this category spans a clinically heterogeneous range and overlaps with cutpoints that changed after TAILORx and RxPONDER. Multiple factors may contribute to the observed trends among intermediate-risk AA women, including differences in tumor biology, treatment adherence, access to care, and broader social drivers of health^18,27–30^. Since its introduction, the Oncotype DX assay has been integral to breast cancer management; however, uncertainty has persisted regarding the overall benefit of chemotherapy for patients with an intermediate-risk recurrence score^31,32^. This uncertainty prompted the development of a randomized trial, TAILORx, to answer this question^33^, and it should be noted that this current cohort of patients was treated prior to the publication of the TAILORx study results. Thus, some of this difference may be explained by differences in chemotherapy receipt in the intermediate risk group. Within the historical RS 19–30 (contemporary RS 16–25) intermediate-risk window specifically, treatment-related confounding is most acute because this is precisely the RS range in which both TAILORx and RxPONDER demonstrated heterogeneous chemotherapy benefit by age, menopausal status, and nodal status; we did not observe similar diagnostic confounding in the low- or high-risk RS groups in the published literature. Further analysis of this intermediate-risk group is constrained by the absence of structured treatment variables in our analytic dataset and by conflicting evidence in the existing literature regarding disparities in chemotherapy receipt following completed Oncotype DX testing, with some studies reporting no disparities and others reporting persistent differences^34,35^. The Oncotype DX assay might be interpreted based on race, socioeconomic status, and availability of treatment modalities, particularly in the intermediate-risk category. Consequently, physician decision-making may reflect an inclination to accommodate patient preferences or the presence of systemic barriers to chemotherapy administration, potentially contributing to a lower likelihood of chemotherapy recommendation for AA patients. Additionally, prior research has documented delays in treatment initiation^36^, lower adherence to endocrine therapy^37–39^, and disparities in healthcare access^40^ among AA patients, all of which can impact long-term breast cancer outcomes. Finally, menopausal status is another key factor in adjuvant chemotherapy decisions. In the TAILORx trial, women over 50 with a Recurrence Score result of 11–25 saw no benefit from adding chemotherapy to endocrine therapy, while those aged 50 or younger with a score of 16–25 experienced a modest benefit^31^. Moreover, the RxPONDER trial found no chemotherapy benefit for postmenopausal women with scores up to 25, whereas premenopausal women showed significant improvements in invasive disease-free and distant relapse-free survival, even at lower scores^12^. While this study adjusted for clinical covariates, unmeasured confounders such as socioeconomic status, insurance coverage, and healthcare provider biases may also play a role in shaping the observed survival patterns. The lack of significant differences in OS and RFS between racial groups suggests that equitable application of clinical management strategies, including the use of adjuvant systemic therapy, may help mitigate potential disparities in outcomes. In a unified health care system where treatment algorithms are consistently applied based on clinicopathologic features and not race or ethnicity, prior studies have demonstrated that these disparities in outcomes are markedly attenuated^41^. Our study further reinforces this as all patients were treated within a single, unified health care system with treatment guidelines applied across all sites. These findings reinforce the critical need to ensure equitable access to guideline-concordant treatment to optimize survival outcomes across all racial groups. Furthermore, the identification of diabetes as a significant predictor of poorer overall survival in our cohort highlights the impact of comorbid conditions on breast cancer prognosis. Since chronic health conditions disproportionately affect African American populations^42,43^, a multidisciplinary approach that incorporates comorbidity management into breast cancer treatment may help reduce disparities and enhance overall patient outcomes.

This study presented a large sample size in real-world clinical settings and provided a robust evaluation of Oncotype DX performance across racial groups. However, it has certain important limitations that we wish to acknowledge transparently. First, the analytic dataset did not include structured, abstracted variables for receipt of adjuvant chemotherapy, radiation therapy, or endocrine therapy, nor for menopausal status. Because these are critical components of breast cancer treatment and known determinants of survival, our multivariable Cox models cannot adjust for them, and we caution the reader that some portion of the residual variation in RFS and OS may be attributable to differences in these treatment-related variables rather than to the prognostic value of the Recurrence Score or to social determinants of health per se. Second, and relatedly, we did not have a usable date of testing/treatment variable in the analytic dataset, which prevented us from conducting the formal pre/post-TAILORx (publication 2018) and pre/post-RxPONDER (publication 2021) sensitivity analyses that would otherwise be informative; we expect that practice-pattern changes after each of these publications meaningfully altered chemotherapy receipt in the intermediate-risk RS window, and a structured pre/post-publication sensitivity analysis remains an important open question that we plan to address in a prospective companion study. Third, because we did not directly measure social determinants of health, including insurance status, area deprivation index, distance to care, employment, transportation, or treatment delay, our discussion of social determinants of health is contextual and drawn from the published literature rather than from empirical analysis of our own cohort. Fourth, the small sample sizes of Asian patients and patients from other racial groups precluded meaningful additional subgroup analyses. Furthermore, the classification of race relied on patient self-reporting, inherently subject to potential inaccuracies and errors. Additionally, the utilization of the Oncotype DX assay may have been influenced by various factors over the decade-long study period, including changes in its availability, accessibility, cost, and adoption rates across different healthcare settings. These evolving factors could contribute to variability in the assay’s application, leading to potential disparities in its under- or overuse that were not directly examined in our analysis.

The published literature consistently describes systemic barriers and social determinants of health that affect treatment outcomes in AA women, including delays in treatment initiation, differences in treatment adherence, and overall access to healthcare, which are known to affect health outcomes significantly. Because the analytic dataset for this study did not include individual-level measurements of social determinants of health (insurance status, area deprivation index, distance to care, employment, transportation, treatment delay), we did not directly demonstrate that the AA women in our cohort experienced these specific barriers; we therefore frame the role of social determinants of health as a hypothesis-generating consideration consistent with the broader literature rather than an empirical finding of our study. Addressing these issues requires a concerted effort to remove barriers to equitable care, ensuring that all patients receive the best possible prognosis and treatment options based on their individual risk assessments. Prospective studies should incorporate detailed analyses of treatment modalities and further explore the biological and socio-environmental factors contributing to disparities in cancer risk stratification. Enhanced understanding through multi-omics approaches could refine the utility of precision medicine tools like the Oncotype DX, ensuring equitable and personalized care across all populations.

AA women with early-stage BC showed a higher prevalence of intermediate- and high-risk Recurrence Score results than White women, which indicates a higher risk of recurrence based on genomic profiling. The overall long-term recurrence-free and overall survival rates were generally similar between the two groups. Importantly, sensitivity analyses using RS 0-25 versus 26–100 and RS 0–15, 16–25, and 26–100 did not identify an independent association between AA race and worse RFS or OS, supporting the prognostic consistency of the Oncotype DX assay across racial groups in this cohort. Nonetheless, a notable exception was observed among women with intermediate risk scores, who had a trend towards worse outcomes compared to their White counterparts. Importantly, the Oncotype DX assay remained a robust prognostic tool for RFS across all racial groups, confirming its reliability in predicting recurrence risk. These findings suggest that while the Recurrence Score results indicate higher risk, they do not fully account for the observed survival outcomes across different racial groups. Other underlying factors and social drivers of health may also contribute to these differences. Further research is essential to refine the application of genomic risk assessment tools such as the Oncotype DX assay in diverse populations, to ensure that they provide equitable benefits and to improve their integration with clinical and demographic factors to enhance personalized, high-quality treatment strategies for all patients.

## Methods

### Study design and patient population

We conducted a retrospective cohort study using data abstracted from the electronic medical record (EMR) at University Hospitals Seidman Cancer Center, a single tertiary academic institution in Cleveland, Ohio. This study was conducted according to the Declaration of Helsinki and was approved by the SpartaIRB, the institutional review board at University Hospitals Seidman Cancer Center, Cleveland, Ohio (STUDY20230927). A full waiver of consent was granted according to the SpartaIRB criteria. Patients were identified via structured EMR queries followed by manual chart review, with Oncotype DX results obtained directly from the clinical Oncotype DX report uploaded to the EMR. The cohort included 1122 women diagnosed with a biopsy-confirmed non-metastatic breast cancer who underwent clinical Oncotype DX testing between January 2013 and September 2023.

### Data collection and variables

Variables captured in structured form in the analytic dataset included age at diagnosis, self-reported race and ethnicity, pathologic T and N stage, tumor size, RS, and a panel of comorbidities. Because the absolute proportion of missingness on these structured variables was small, a complete-case analysis was performed for the multivariable models. We acknowledge that receipt of adjuvant chemotherapy, radiation therapy, and endocrine therapy, as well as menopausal status, were not consistently captured in structured form in the analytic dataset, and these clinically important variables could not be included in the multivariable models. We have highlighted this as a primary limitation in the Discussion. Patients were categorized into four different groups based on their self-reported race: African American (AA), White, Asian, and other racial groups. Asian and other racial groups were excluded from survival analyses because of the groups’ small sample sizes.

### Oncotype DX risk stratification

Recurrence Score® results were initially classified into three risk categories: low (0–18), intermediate (19–30), and high (31–100). These historical cutpoints reflect the standard reporting and clinical decision framework that was used during the majority of the study period. To address contemporary clinical practice as guided by TAILORx and RxPONDER, we pre-specified two contemporary-cutpoint sensitivity analyses: a binary scheme (RS 0–25 vs RS 26–100) and a three-level scheme (RS 0–15, 16–25, 26–100). Because menopausal status was not consistently captured in the analytic dataset, the contemporary cutpoints were applied uniformly across the cohort rather than stratified by menopausal status. The contemporary-cutpoint sensitivity analyses parallel the primary historical-cutpoint analysis (descriptive distribution by race; Kaplan-Meier RFS and OS; multivariable Cox models) and are reported in Tables 5–10.

### Study objectives

The primary objective was to investigate the association between Oncotype DX test results and survival outcomes. The secondary objectives include investigating racial disparities in the distribution of Oncotype DX results and identifying demographic and clinical factors that may influence the relationship between genomically stratified molecular test results and outcomes.

### Outcomes and statistical analysis

Patients and tumor characteristics were summarized using descriptive statistics. Continuous variables were reported using mean and standard deviation, while categorical variables were reported using counts and percentages. Recurrence-free survival (RFS) was defined as the time from the date of primary diagnosis to the earliest occurrence of locoregional recurrence or death from any cause. Patients who were lost to follow-up before the maximum observation time of 10.75 years or who remained recurrence-free at the end of the study period were censored at the date of last contact or at the maximum observation time, whichever came first. Overall survival (OS) was defined similarly using the date of death. Survival outcomes, including OS and RFS, were estimated using the Kaplan-Meier method. Multivariable Cox proportional hazards models were used to adjust for potential confounders (age, tumor stage, hormone receptor status). Interaction terms between race and Oncotype DX score were evaluated using a likelihood ratio test. The proportional hazards assumption was assessed using Schoenfeld residuals. Statistical analyses were performed using R statistical software, version 4.4.1 (R Foundation for Statistical Computing, Vienna, Austria). A two-tailed *p*-value of less than 0.05 was considered statistically significant.

## Supplementary information

Supplementary tables.

## Data Availability

The datasets generated and/or analyzed during the current study are not publicly available due to the large size and complexity of the dataset but are available from the corresponding author on reasonable request.
