# Supplementary material for: Prognostic consistency of oncotype DX® molecular assay across racial groups in early-stage hormone receptor-positive breast cancer
Source: NPJ Breast Cancer. 2026 Jun 25;12:133. doi: 10.1038/s41523-026-00996-z (PMC13658793; doi:10.1038/s41523-026-00996-z)
Supplement: Supplementary file 1 [file 41523_2026_996_MOESM1_ESM.pdf]

Table S1. Patients' demographics and characteristics. Abbreviations: SD, standard deviation. Percentages may not sum to 100% because of rounding and missing data.

	Patients N=1122
Age at diagnosis, mean (SD)	60.8 (10.5)
T stage	
T0-1	782 (69.7%)
T2	307 (27.4%)
T3-4	18 (1.6%)
Missing data	15 (1.3%)
N stage	
N0(i+)	35 (3.1%)
N0	879 (78.3%)
N1	175 (15.6%)
N2	4 (0.4%)
Nx	14 (1.2%)
Missing data	15 (1.34%)
Comorbidities	
Cardiac	155 (13.8%)
Pulmonary	82 (7.3%)
Diabetes	57 (5.1%)
Hypertension	101 (9.0%)
Obesity	32 (2.9%)
Other cancers	390 (34.8%)
Gastrointestinal	58 (5.2%)
Renal	40 (3. 6%)
Other comorbidities	956 (85.2%)

Table S2. RFS and OS estimates stratified by racial group. Values are Kaplan-Meier estimates reported as percentages with 95% confidence intervals. Abbreviations: AA, African American; RFS, recurrence-free survival; OS, overall survival.

Race		AA	White
RFS	3-year	94.1 (90.0-98.5)	95.0 (93.5-96.5)
	5-year	90.7 (85.2-96.5)	90.4 (88.3-92.6)
	10-year	76.8 (65.9-89.5)	74.6 (68.5-81.2)
OS	3-year	98.3 (96.0-100)	97.6 (96.6-98.7)
	5-year	94.0 (89.3-98.8)	93.2 (91.4-95.1)
	10-year	78.2 (65.7-93.1)	74.8 (68.4-81.8)
